# Susceptibility Assessment of Glacier-Related Debris Flow in the Gaizi River Basin Using Different Hybrid Anomaly Detection Models

**DOI:** 10.3390/s26123884

**Published:** 2026-06-18

**Authors:** Wentao Cheng, Tie Liu, Yue Huang, Weiyi Mao, Anming Bao, Yousef A. Al-Masnay, Peng Du, Zhiyong Zhang, Ying Liu

**Affiliations:** 1 State Key Laboratory of Ecological Safety and Sustainable Development in Arid Lands, Xinjiang Institute of Ecology and Geography, Chinese Academy of Sciences, Urumqi 830011, China; chengwentao24@mails.ucas.ac.cn (W.C.); huangy@ms.xjb.ac.cn (Y.H.); baoam@ms.xjb.ac.cn (A.B.); yousefmasnay@mails.ucas.ac.cn (Y.A.A.-M.); 2 University of Chinese Academy of Sciences, Beijing 100049, China; 3 Key Laboratory of RS & GIS Application, Xinjiang Uygur Autonomous Region, Urumqi 830011, China; 4 College of Geoinformatics, Zhejiang University of Technology, Hangzhou 310014, China; liutie@zjut.edu.cn; 5 Institute of Desert Meteorology, China Meteorological Administration, Urumqi 830002, China; 6 Xinjiang Uygur Autonomous Region Natural Disaster Comprehensive Monitoring and Early Warning Center, Urumqi 830011, China; yq258@gmail.com (P.D.);

**Keywords:** glacier-related debris flow, susceptibility assessment, hybrid model, anomaly detection, Gaizi River basin

## Abstract

**Highlights:**

**What are the main findings?**

- Topographic factors are the primary indicators for assessing the susceptibility of glacier-related debris flows in the Gaizi Basin. In contrast, distance to glacier and precipitation appear less informative for direct susceptibility inference under our specific dataset and analytical setup.
- Whereas other anomaly detection models tend to overestimate susceptibility, GAN’s architecture produces more balanced results.

**What are the implications of the main findings?**

- Key factors for assessing glacier-related debris flows: distance to stream, slope, topographic roughness/wetness indices, and solar radiation.
- When high-quality multi-label susceptibility data are unavailable, one-class anomaly detection models can be applied using a disaster inventory.

**Abstract:**

The Gaizi River Basin, an alpine region in China crossed by the Karakoram Highway, is highly prone to glacier-related debris flows (GDF). Accurate debris flow susceptibility assessment in this high-altitude area remains challenging due to complex terrain, active tectonics, and dynamic glacial processes. This study develops a hybrid model integrating statistical methods and machine learning-based anomaly detection for debris flow susceptibility mapping. To address data noise, certainty factor (CF) distributions of debris flow predisposing factors (DFPFs) were derived via Locally Weighted Scatterplot Smoothing (LOWESS). The strength of the association between DFPFs and GDF susceptibility was evaluated using the mean residual between the raw and LOWESS-smoothed CF values. Multiple anomaly detection algorithms, including distance-based (L2 Norm), density-based (One-Class SVM), ensemble (Isolation Forest, RandNet), and GAN-based (WBiGAN-GP) methods, were tested on raw and CF-transformed data, using only the GDF inventory as the label. The CF-WBiGAN-GP model delivers the most balanced performance, excelling at identifying both high- and low-susceptibility zones. Results show that distance to stream, slope, and the topographic roughness and wetness indices are strongly associated with GDF susceptibility. Distance to glacier and precipitation appear less informative for direct susceptibility inference under our specific dataset and analytical setup.

## 1. Introduction

The Gaizi River Basin, a high-altitude region traversed by the Karakoram Highway (KKH), the primary land corridor between China and Pakistan, is frequently affected by glacial debris flows (GDF). The area is characterized by the rapid mobilization of loose debris, significantly influenced by glacial meltwater and seasonal rainfall [1]. About 200 km section of KKH through the basin is facing severe threats from these recurrent events. Therefore, the susceptibility assessment of glacier-related debris flows for the Gaizi River Basin is crucial to provide essential insights for safeguarding this vital infrastructure and to support the secure planning of subsequent infrastructure projects along the China–Pakistan Economic Corridor within the basin. However, accurately assessing debris flow susceptibility in this high-altitude region remains challenging due to complex terrain, active tectonics, and glacial processes.

In debris flow susceptibility assessment, three types of methods are commonly employed: semi-empirical models, statistical models, and machine learning models. Semi-empirical models, grounded in physical experiments, offer the highest interpretability [2,3]; however, their idealized parameters often fail to adequately represent complex real-world conditions, limiting their predictive performance. Statistical models, which utilize statistical techniques to identify relationships between variables (e.g., certainty factors, factor weight), offer improved adaptability to empirical data compared to semi-empirical methods, though they tend to be heavily data-dependent. Machine learning models (e.g., LR, RF, XGBoost), while often treated as “black-box” systems due to their low interpretability, contain numerous parameters that enable them to capture complex coupling effects among DFPFs, generally yielding superior predictive results [4,5]. Nevertheless, the performance of machine learning models is heavily reliant on the quality of input data, with issues such as incompleteness, noise, or bias directly affecting model stability and generalizability. Thus, hybrid models integrating statistical and machine learning methods have gained attention for their potential to enhance the robustness and accuracy of debris flow susceptibility assessments [6].

A common practice in constructing the models and presenting the results of debris flow susceptibility assessment involves classifying both factor values and susceptibility levels within the study area. This approach, however, introduces subjectivity in standardizing classification criteria, and ambiguous boundary definitions, limiting its generalizability. To minimize noise and subjectivity, the Certainty Factor (CF) distributions obtained through Locally Weighted Scatterplot Smoothing (LOWESS) of each factor were used in this paper. Meanwhile, anomaly detection algorithms were applied for the debris flow susceptibility assessment by taking the debris flow inventory as the high-susceptibility zones.

The selection of appropriate debris flow predisposing factors (DFPFs) is another critical aspect of debris flow assessment which directly influences the accuracy of debris flow susceptibility predictions. Building upon previous research on analogous GDF events [6,7,8], this study systematically selected five categories of DFPFs to characterize the initiation mechanisms of glacial debris flows: meteorological, hydrological, topographical, geological, and land cover conditions.

This study aims to develop a hybrid modeling framework that integrates statistical models based on CF and LOWESS with multiple anomaly detection algorithms. Under the constraint of limited labeled data, we used only the debris flow inventory as the training label to achieve susceptibility mapping of GDFs.

## 2. Materials and Methods

### 2.1. Study Area

**Figure 1:**
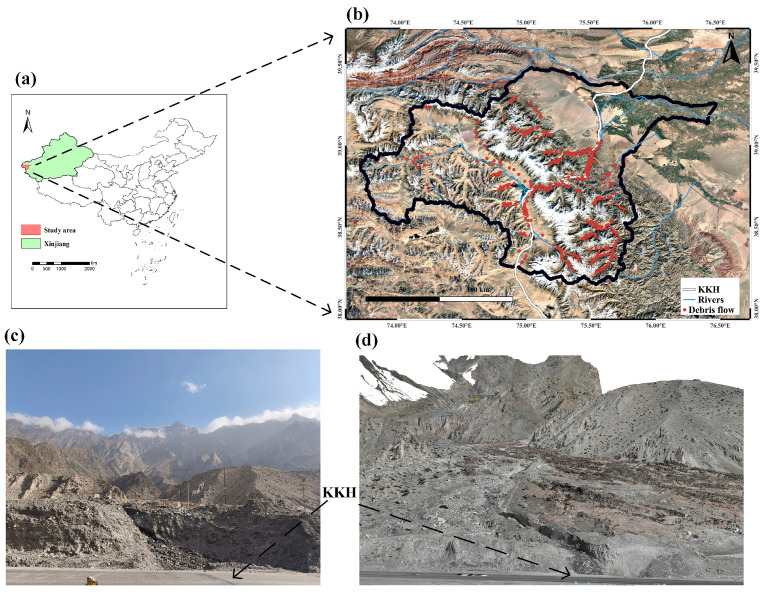
Location and topographic map of the study area. (**a**) The map shows the location of Gaizi River basin. (**b**) Debris flows inventory map. (**c**,**d**) Representative field photographs and three-dimensional channel models of the debris flow gullies.

The Gaizi River Basin (73°42′1″ E–76°29′40″ E, 38°9′31″ N–39°29′13″ N) is a typical high-altitude mountainous region situated in the northern Karakoram, covering a total area of approximately 17,963.70 km^2^, as shown in Figure 1. The basin exhibits remarkable topographic heterogeneity, with elevations ranging dramatically from 1209 m to 7629 m above sea level, and an average elevation of 3550.7 m. This extreme relief, combined with the region’s complex geological structure, creates favorable conditions for various geomorphological processes and associated hazards. The region’s hydrology is dominated by glacial meltwater and seasonal precipitation. Geologically, the basin is situated in a tectonically active zone where the convergence of the Indian and Eurasian plates has created complex fault systems and frequent seismic activity, further destabilizing slopes and contributing to sediment availability.

The Karakoram Highway (KKH) is the only overland transportation route connecting China and Pakistan. This vital artery begins at Kashgar, follows the Gaizi River valley, passes through the Khunjerab Port, and extends into Pakistan, serving as a critical transportation link between the two nations and a key component of the Belt and Road Initiative. The approximately 200-km section of the KKH traversing the Gaizi River Basin experiences recurrent disruptions due to debris flows and other geological hazards [3,9]. The area demonstrates particular susceptibility to debris flows, which are triggered by a combination of DFPFs including glacial meltwater, seasonal rainfall, abundant loose sedimentary material, and steep topography. These events pose severe threats to transportation safety, infrastructure integrity, and socioeconomic activities along this critical corridor. Consequently, the Gaizi River Basin has emerged as a priority area for geohazard susceptibility research, disaster risk reduction strategies, and sustainable highway maintenance planning, representing a compelling case for geohazard susceptibility research in high-mountain environments.

### 2.2. Datasets

**Figure 2:**
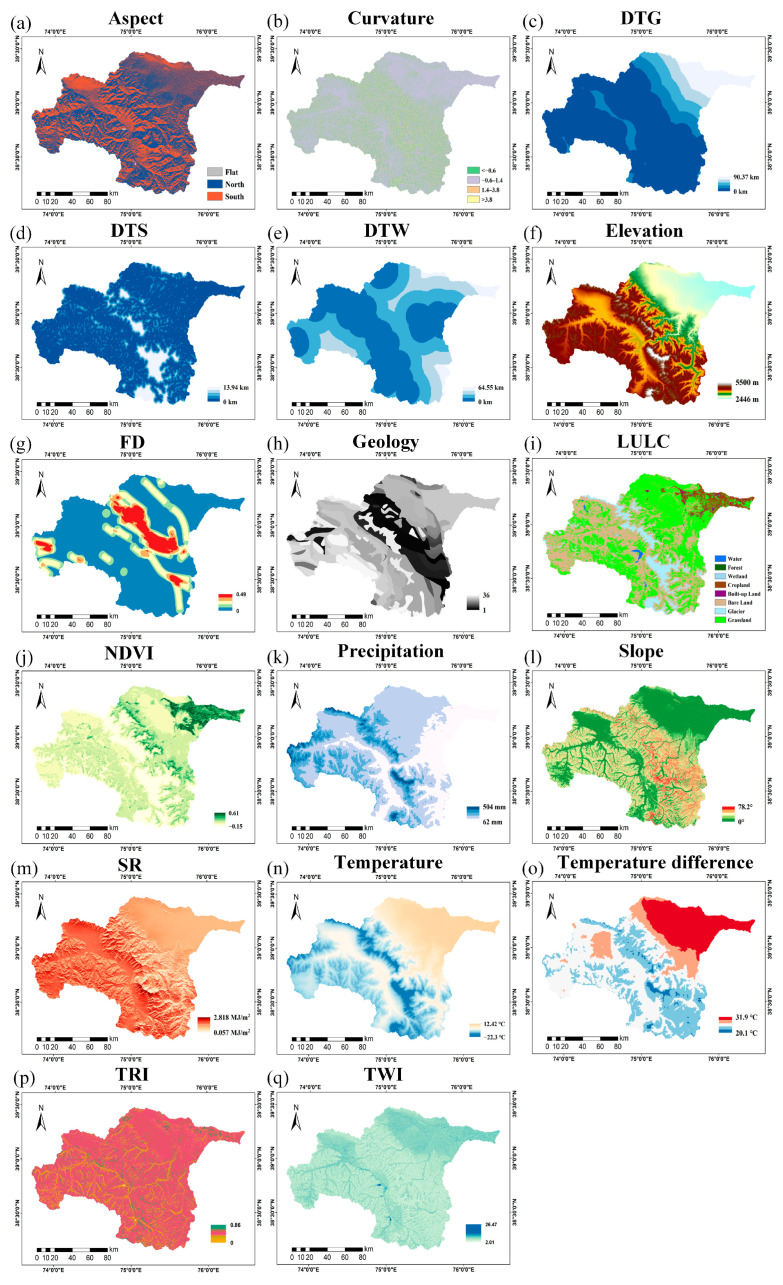
Spatial distribution of 17 DFPFs. (**a**) aspect; (**b**) curvature; (**c**) distance to glacier; (**d**) distance to stream; (**e**) distance to water body; (**f**) elevation; (**g**) fault density; (**h**) geology; (**i**) land use and land cover; (**j**) NDVI; (**k**) precipitation; (**l**) slope; (**m**) solar radiation; (**n**) annual average temperature; (**o**) annual temperature difference; (**p**) topographic roughness index; (**q**) topographic wetness index.

Seventeen DFPFs were selected for debris flow susceptibility modeling based on existing data availability, field surveys, and their recognized influence on debris flow initiation mechanisms in high-mountain glacial environments [3,6]. These factors were categorized into five groups: topographic, hydrological, meteorological, land cover, and geological factors, as shown in Table 1 and Figure 2. The high-susceptibility zones were derived from the debris flow inventory. To validate model performance, low-susceptibility zones were incorporated into the assessment.

#### 2.2.1. Topographic Factors

Topographic factors govern the gravitational and geomorphic processes central to debris flow initiation and propagation. Elevation influences local climate conditions, material transport capacity, and the distribution of erosion processes. Slope directly controls gravitational driving forces and surface water velocity, governing sediment entrainment and transport capacity [14]. Aspect modulates microclimatic conditions by regulating solar exposure, thereby affecting soil moisture, weathering rates, and vegetation patterns. Curvature quantifies surface convergence and divergence, influencing runoff concentration and sediment deposition patterns. Topographic Roughness Index (TRI) characterizes surface irregularity, which affects flow resistance and sediment retention capacity. Topographic Wetness Index (TWI) models spatial saturation patterns, indicating potential zones for soil saturation and pore pressure development [15].

#### 2.2.2. Hydrological, Meteorological and Land Cover Factors

These categories encompass dynamic and surface-cover variables that directly or indirectly regulate water availability, slope stability, and material mobility. Distance to Glacier (DTG) reflects proximity to glacial meltwater sources, a key trigger mechanism in high-mountain environments. Distance to Stream (DTS) indicates susceptibility to channel erosion and bank collapse processes. Distance to Water Bodies (DTW) integrates overall hydrological influence, including potential groundwater effects. Precipitation represents the triggering mechanism through infiltration and runoff generation. Annual Average Temperature (Temperature) influences permafrost distribution, weathering rates, and long-term glacier equilibrium. Annual Temperature Difference indicates seasonal variability that controls freeze–thaw cycling and meltwater regime intensity. Solar Radiation (SR) drives evapotranspiration and snowmelt processes, affecting soil moisture conditions. Normalized Difference Vegetation Index (NDVI) quantifies vegetation density, which modulates slope stability through root reinforcement and hydrological regulation. Land Use and Land Cover (LULC) represents human and natural modifications to surface conditions that affect infiltration capacity and erosion susceptibility.

#### 2.2.3. Geological Factors

Geological factors determine the inherent susceptibility of slope materials to failure and erosion. Fault Density (FD) delineates zones of structural weakness where rock mass integrity is compromised, facilitating material mobilization. Geology determines fundamental geotechnical properties through lithological composition and rock mass characteristics that govern material strength and erosion resistance.

#### 2.2.4. Debris Flow Inventory and Susceptibility Zoning

**Figure 3:**
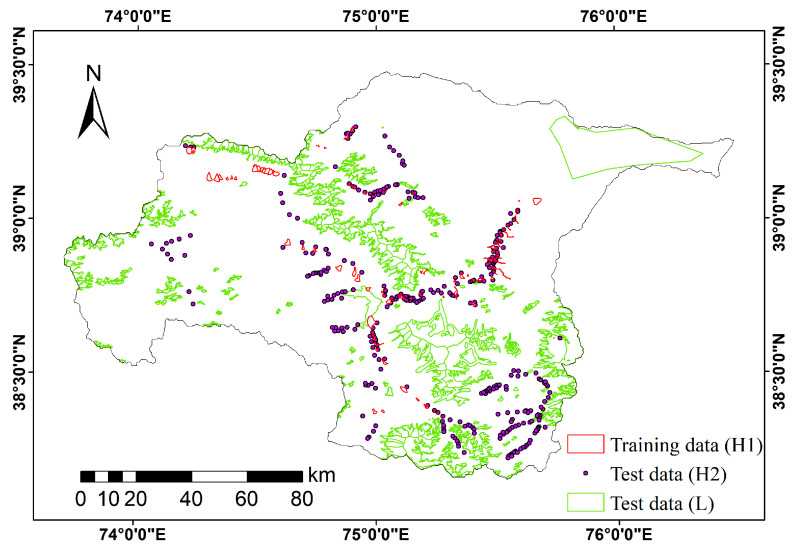
Training data and test data of this study.

As shown in Figure 3, the training data (H1) comprises 122 glacier-related debris flow zones identified through remote sensing interpretation for model training. The test data (H2) consists of an additional 416 glacier-related debris flow sites obtained from the published inventory [1] for independent validation. These documented areas, representing locations with clear evidence of past debris flow activity, were utilized as positive samples throughout the modeling process. To ensure a rigorous validation of model performance, low-susceptibility zones (L), comprising flat urban residential areas in Akto County and glacier/lake areas in the Gaizi Basin, labeled as negative samples, were incorporated to evaluate model performance.

### 2.3. Methods

**Figure 4:**
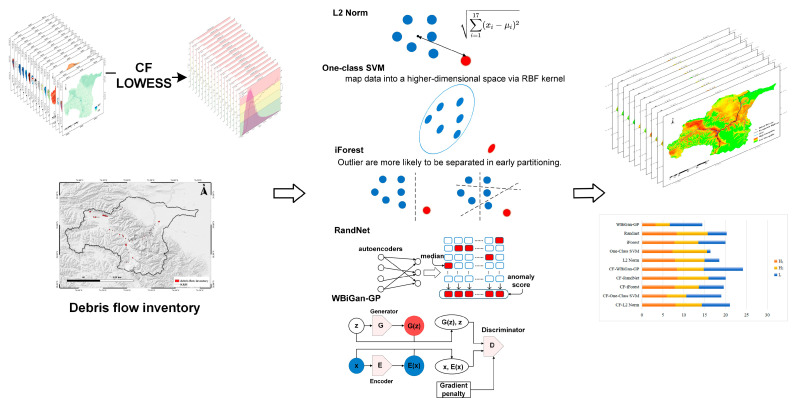
Flowchart of this study.

The flowchart of this study, shown in Figure 4, involves four main parts: Firstly, a debris flow inventory was established by visual interpretation to delineate high-susceptibility zones and 17 DFPFs were systematically collected for the study area. Secondly, raw factor values were discretized into bins through statistical modeling and converted to Certainty Factor (CF) values and were subsequently transformed into continuous distributions using LOWESS. Thirdly, machine learning models were applied by feeding both raw and CF-transformed data into five distinct anomaly detection algorithms (L2 Norm, One-Class SVM, iForest, RandNet and WBiGAN-GP) to generate the susceptibility index (SI). Finally, not only high-susceptibility areas but also low-susceptibility zones were incorporated to comparatively assess and verify the predictive effectiveness and discrimination capability of models.

#### 2.3.1. Statistical Models

The certainty factor (CF) [16] can be calculated by Equation (1):

(1) $$CF=\left\{\begin{array}{c} \frac{PP_{a}-PP_{s}}{PP_{a}(1-PP_{s})}\;\;\mathrm{if}\;PP_{a}\geq PP_{s}\\ \frac{PP_{a}-PP_{s}}{PP_{s}(1-PP_{a})}\;\;\mathrm{if}\;PP_{a}<PP_{s} \end{array}\right.$$

where PP_a_ is the conditional probability of debris flow occurrence within class a, PP_s_ is the prior probability of debris flow occurrence across the entire study area (the overall probability of debris flow events).

The CF value ranges from −1 to 1. A positive value indicates an increase in the certainty of debris flow occurrence, a negative value signifies a decrease in the certainty of debris flow occurrence, and a value close to 0 suggests that the variable provides insufficient information regarding debris flow occurrence.

A data-driven and robust continuous distribution of CF values is required to objectively represent the relationship between each predisposing factor and debris flow susceptibility. However, the factor values derived from sampled high-susceptibility areas are inherently discrete and incomplete. Operating on the premise that the variation in CF values is spatially smooth rather than abrupt, this study adopted the Locally Weighted Scatterplot Smoothing (LOWESS) method to generate continuous and robust CF curves [17].

LOWESS is a non-parametric regression technique that fits a smooth curve to a set of data points without assuming a fixed global functional form (e.g., linear or polynomial). Instead, it models complex trends by constructing a series of local approximations.

The core of the LOWESS algorithm involves defining a local neighborhood, or bandwidth, which specifies the number of adjacent bins used for each local regression. For a target point within this bandwidth, a weight is assigned to each bin. The weighting scheme, formalized in Equation (4), ensures that the weight of a bin is inversely related to its distance from the target and positively related to its local data size. The distance signifies the degree of similarity, whereas the data size reflects the confidence level in the corresponding CF value. During the initial iteration, the tri-cube weight function was employed. In subsequent iterations, a standardized residual term was incorporated to reduce the influence of outliers (i.e., points with large residuals), thereby enhancing the robustness of the smoothed results. The CF value at the target point is then obtained by solving the local weighted least squares problem defined in Equation (5). This process is iterative; for each iteration, the model parameters are refined to minimize the residuals. By sliding the bandwidth across the entire factor range and repeating this local regression, a smooth and continuous CF curve is generated for each DFPF.

(2) $$u_{i}=\frac{1}{2}(\frac{i}{h}+1-\frac{n_{i}}{\sum_{i}^{t}n_{i}})$$

(3) $$s_{i}=\frac{y_{i}-\hat{y_{i}}}{\left(6\times MAD\right)}$$

(4) $$w_{i}=\left\{\begin{array}{ll} {\left(1-u_{i}^{3}\right)}^{3} & \mathrm{if}\;t=1\\ {\left(1-s_{i}^{2}\right)}^{2}{\left(1-u_{i}^{3}\right)}^{3} & \mathrm{if}\;t>1 \end{array}\right.$$

(5) $$\min\sum_{i}^{h}w_{i}{\left(\alpha x_{i}-y_{i}\right)}^{2}$$

where h denotes the bandwidth and i is the index of a specific bin within it. For the i-th bin, nᵢ is the data size, MAD denotes the median absolute deviation of the residuals and the coefficient 6 ensures that approximately 99% of normally distributed data fall within the range, w_i_ is the local weight, x_i_ is the raw factor value, and yᵢ is the corresponding CF value. Additionally, t represents the iteration counter and α is a constant.

In the experiments, the maximum number of bins was initially set for each continuous factor. Bins with no data or insufficient data points were deemed unreliable and thus discarded. It should be noted that the Geology and LULC are inherently discrete and therefore were not transformed by LOWESS. To address the uneven density of data points across different value ranges, where some DFPFs (e.g., Aspect) are uniformly distributed while others (e.g., Distance to Stream (DTS), Distance to Glacier (DTG), and Fault Density (FD)) are highly clustered, the value range of each factor was divided into different bins. The bin count was determined adaptively based on the distribution characteristics within the high-susceptibility areas, with a principle of selecting a sufficiently large number of bins to approximate a continuous distribution. The corresponding experimental parameters are provided in Table 2. The iteration count primarily addresses outliers to improve the model’s robustness. In contrast, the bandwidth parameter manages the smoothness, intrinsically embodying a bias-variance trade-off. The LOWESS parameters were chosen empirically based on the results, as they are difficult to evaluate using quantitative metrics. Specifically, the window ratio ranged from 0.1 to 0.7 (bandwidth = window ratio × bins), iterations from 1 to 6, and bin sizes were 50, 75, 100, and 200.

#### 2.3.2. Anomaly Detection Models

This paper employed five anomaly detection methods to evaluate the susceptibility of glacier-related debris flows. The methods were categorized into four distinct paradigms: a distance-based approach implemented using the L2 Norm (Euclidean distance); a density-based approach represented by One-Class SVM; ensemble methods, including Isolation Forest (iForest) and RandNet; and a generative adversarial network (GAN)-based method, specifically WBiGAN-GP. The susceptibility index was obtained by applying linear transformation to the anomaly scores derived from anomaly detection methods, normalizing them to a [0, 1] range across the study area. This technique assigned a specific risk value to each location, achieving high spatial granularity. The main parameters are listed in Table 3.

The L2 Norm, or Euclidean distance, was employed as a fundamental distance-based anomaly scoring method to establish a geometric and intuitive benchmark for debris flow susceptibility. This approach operates on the premise that the debris flow inventory defines the feature space of high-susceptibility conditions. Therefore, areas exhibiting high susceptibility patterns will be positioned close to the centroid of this normative environment (debris flow inventory), while areas positioned far from this centroid in the multi-dimensional feature space are identified as anomalies, indicating low susceptibility. A higher L2 Norm score indicates a greater deviation from the average conditions, which we interpret as a higher likelihood of being an anomalous, low-susceptibility zone.

The One-Class Support Vector Machine (One-Class SVM) [18,19] was implemented as a density-based anomaly detection method to identify regions of high debris flow susceptibility. Unlike supervised classifiers that require data from all classes, One-Class SVM is designed for scenarios where only the distribution of the “normal” or target class is available for training, making it ideally suited for our objective of modeling based solely on known high-susceptibility areas. The Radial Basis Function (RBF) kernel was selected for its ability to capture complex, non-linear relationships between DFPFs in high-dimensional space. In this study, the algorithm is configured to expect approximately 10% of the training data (high-susceptibility samples) to be potential outliers, thereby enforcing a tighter and more conservative decision boundary.

The Isolation Forest (iForest) algorithm [20,21] was employed as an efficient, tree-based ensemble method for anomaly detection. Its core principle is based on the concept that anomalous data points, those representing low susceptibility in our context, are inherently “few and different” and thus require fewer random partitions to be isolated from the bulk of the data. The method operates by constructing multiple binary decision trees where data is recursively partitioned through randomly selected features and split values. Anomalous samples, characterized by their distinctive feature values, typically require fewer partitions to be isolated, resulting in shorter path lengths from the root to the leaf node. The final anomaly score is derived as a function of the average path length across all trees in the ensemble. Consistent with the approach used in our One-Class SVM implementation, we explicitly defined the expected proportion of anomalies in the dataset by setting the contamination parameter to 10%, which guides the algorithm to identify the top 10% of the most readily isolatable samples as low susceptibility candidates.

The RandNet Ensemble method [22] was implemented as a deep anomaly detection method, specifically designed to capture complex patterns in debris flow susceptibility data through an ensemble of randomly connected autoencoders. An autoencoder is a multi-layer neural network characterized by its fully connected, symmetric architecture with a central bottleneck hidden layer. The primary goal of an autoencoder is to reconstruct its input as accurately as possible, thereby forcing the network to learn a meaningful and efficient representation of the underlying patterns. There is no guarantee that the combination of multiple detectors will always perform better than the best individual detector in the ensemble. In order to make ensemble learning methods work, the individual ensemble components must be adequately diverse. This is achieved by creating predictive models such that each ensemble component is able to capture different parts of the underlying patterns. The core idea behind introducing random variations in the connectivity architecture of the autoencoders is to achieve significantly better performance. In this study, we implemented a RandNet architecture comprising 21 autoencoders arranged in a five-layer hierarchical structure with a (5, 4, 3, 4, 5) configuration. The final reconstruction is obtained through median aggregation of outputs from all pathways, providing robust anomaly scores for susceptibility assessment.

The WBiGAN-GP framework implemented in this study represents a strategic integration of the Bidirectional Generative Adversarial Network (BiGAN) [23] architecture with the training stabilization techniques of Wasserstein GAN with Gradient Penalty (WGAN-GP) [24]. As shown in Figure 4, the BiGAN component incorporates a unique triad architecture consisting of an encoder (E), generator (G), and discriminator (D). The encoder E maps input data x to latent representations z, while the generator G performs the inverse mapping from latent space to data space. The discriminator D operates jointly in both data and latent spaces, distinguishing between real tuples (x, E(x)) and generated tuples (G(z), z). This bidirectional architecture compels the encoder and generator to learn mutually invertible mappings, thereby achieving the ultimate objective: x ≈ G(E(x)) and z ≈ E(G(z)), while simultaneously reaching a Nash equilibrium with the discriminator [25]. However, our preliminary experiments revealed that the standalone BiGAN architecture suffered from mode collapse, where an overpowered discriminator dominated the training process, resulting in limited diversity in generated samples and compromised feature learning [26]. To address this limitation, we incorporated the WGAN-GP stabilization mechanism, which replaces the original Lipschitz constraint enforcement with a gradient penalty term applied to random interpolates between real and generated data distributions.

The effectiveness of the susceptibility map was evaluated through two specialized metrics designed to measure performance in high-susceptibility and low-susceptibility zones respectively. H quantifies model performance in correctly identifying high susceptibility zones from the debris flow inventory. L evaluates model performance in correctly classifying low-susceptibility zones, with test samples drawn from densely populated regions where debris flow occurrence is highly improbable. H and L metrics are the mean predicted susceptibility indices over positive and negative test points, scaled to a range of [0, 10], computed from Equation (6) and (7) respectively:

(6) $$H=\frac{10}{n_{h}}\sum_{i=1}^{n_{h}}\hat{y_{i}}$$

(7) $$L=\frac{10}{n_{l}}\sum_{i=1}^{n_{l}}\left(1-\hat{y_{i}}\right)$$

where $n_{h}$ and $n_{l}$ represent the number of samples in the high-risk and low-risk test sets, respectively, and $\hat{y}$ is the susceptibility index (SI) predicted by the model.

This study exclusively utilizes high-susceptibility zones derived from the debris flow inventory. While conventional methods often classify susceptibility into three to five categories, requiring a corresponding number of thresholds, this work designs a binary classification scheme for illustrative purposes. In this scheme, regions with SI in the interval (y, 1] are classified as high-susceptibility zones, and those with SI in [0, x) are classified as low-susceptibility zones. Based on this dichotomous division, performance metrics including accuracy, precision, recall, and F1-score are calculated using the labeled high- and low-susceptibility areas.

## 3. Results

### 3.1. CF Distribution of DFPFs

**Figure 5:**
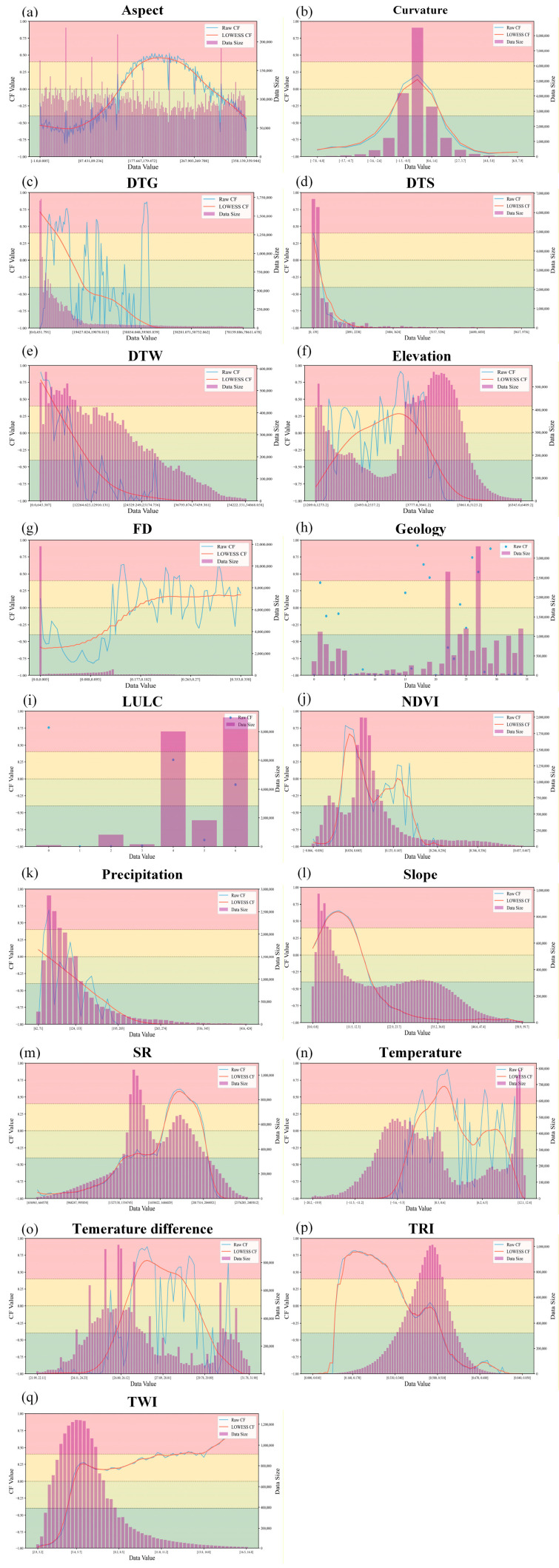
CF distribution of 17 DFPFs. (**a**) aspect; (**b**) curvature; (**c**) distance to glacier; (**d**) distance to stream; (**e**) distance to water body; (**f**) elevation; (**g**) fault density; (**h**) geology; (**i**) land use and land cover; (**j**) NDVI; (**k**) precipitation; (**l**) slope; (**m**) solar radiation; (**n**) annual average temperature; (**o**) annual temperature difference; (**p**) topographic roughness index; (**q**) topographic wetness index.

The CF distribution for each DFPF was shown in Figure 5. The CF distributions of DFPFs, including Aspect, Slope, SR, TRI, and TWI, exhibit good fitting performance, indicating either high data quality with low noise, or a relatively deterministic influence on debris flow occurrence that is independent of other DFPFs.

### 3.2. GDF Susceptibility Map

**Figure 6:**
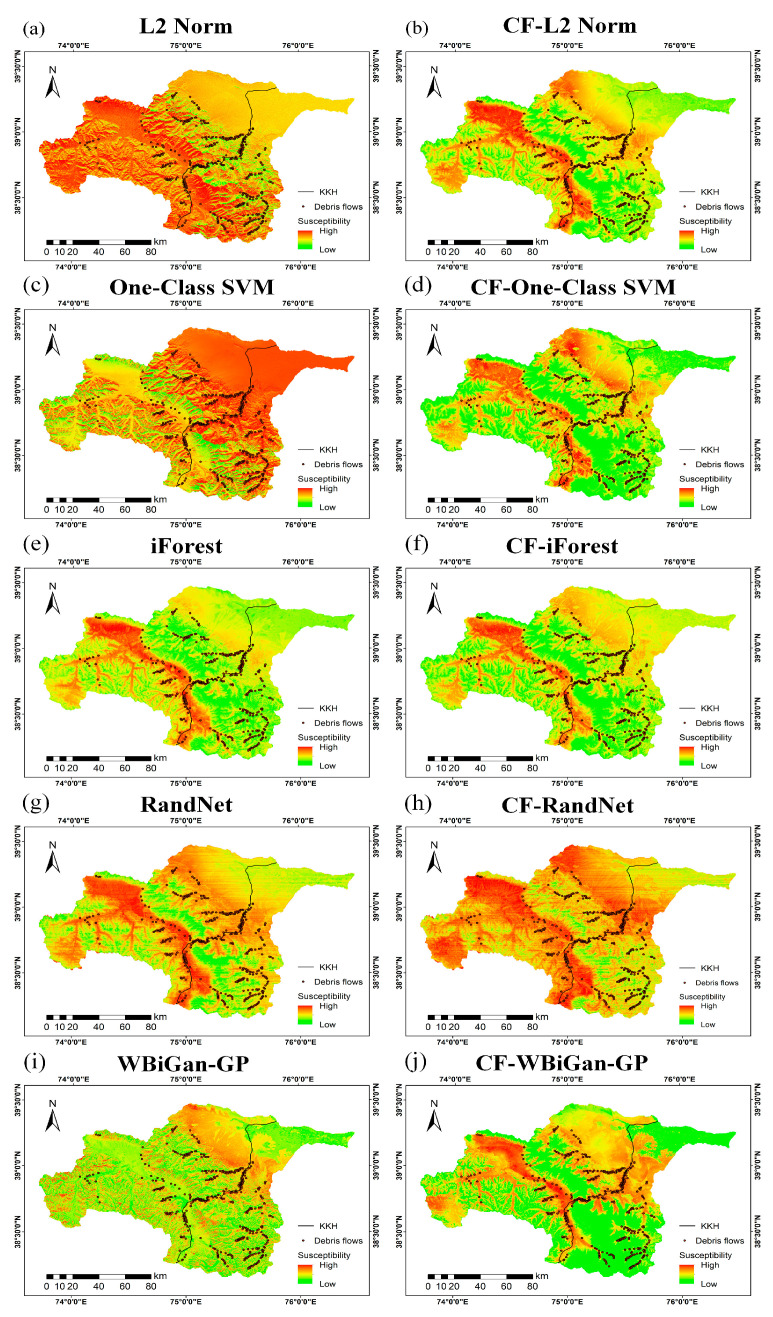
GDF susceptibility map for all applied models. (**a**) L2 Norm; (**b**) CF-L2 Norm; (**c**) One-Class SVM; (**d**) CF-One-Class SVM; (**e**) iForest; (**f**) CF-iForest; (**g**) RandNet; (**h**) CF-RandNet; (**i**) WBiGan-GP; (**j**) CF-WBiGan-GP.

The GDF susceptibility maps are presented in Figure 6. For each grid cell, the GDF susceptibility is represented by the SI output from the models, which can be classified into levels according to actual conditions, e.g., [0.8, 1] denoting very high susceptibility.

The high-susceptibility areas predominantly align with glacial frontal margins, valley directions, and gully systems [27]. The KKH, constructed along these valleys, consequently traverses multiple high-risk sections, creating significant infrastructure vulnerability. Conversely, low-susceptibility areas are primarily concentrated in northeastern urban zones, high mountain ridges, and central glacier regions. However, an inherent limitation of the single-class anomaly detection approach emerges as a systematic tendency toward overestimating susceptibility. This methodological bias results from training exclusively on high-susceptibility samples from the debris flow inventory, causing the models to prioritize sensitivity to potential hazards over specificity in stable area identification.

### 3.3. Model Performance

#### 3.3.1. Model Performance Based on Evaluation Metrics

The performance metrics (H1, H2, L) for all models evaluated in this study are summarized in Table 4. H1 and H2 were computed using Equation (6) to assess model performance in high-susceptibility zones, while L was calculated with Equation (7) to evaluate performance in low-susceptibility zones. Specifically, H1 was computed using 122 debris flow zones from the training set and H2 was derived from a set of 416 debris flow sites from the published inventory for independent validation. L was calculated based on stable urban and glacier/lake areas.

The performance evaluation of anomaly detection models reveals distinct patterns in their ability to identify high- and low-susceptibility zones. CF-WBiGAN-GP demonstrates the most balanced performance overall, achieving excellent scores in all metrics, indicating strong capability in recognizing both hazardous and stable areas. Nevertheless, we acknowledge that the H2 score of CF-WBiGAN-GP is modest, while CF-One-Class SVM, CF-L2 Norm, RandNet, and CF-iForest also perform well, particularly in high-susceptibility areas. CF-RandNet and RandNet demonstrated superior performance in assessing high-susceptibility zones but showed limited effectiveness in evaluating low-susceptibility areas, suggesting a trade-off between sensitivity and specificity.

Comparatively, models utilizing CF-transformed input generally outperform their raw-data counterparts, particularly evident in the WBiGAN-GP and One-Class SVM pairs. This underscores the value of the CF transformation in enhancing feature representation.

The results suggest that while some models excel in detecting high-susceptibility zones (high H1 and H2), they may struggle with accurately classifying stable areas (low L), and vice versa. This performance underscores the importance of evaluating both metrics simultaneously when selecting an optimal model for comprehensive debris flow susceptibility assessment.

**Figure 7:**
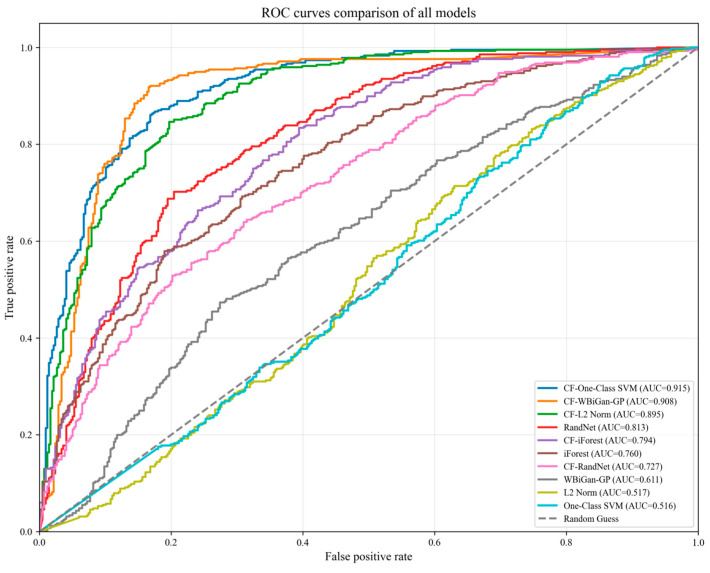
ROC curves and AUC values of all models.

As shown in Figure 7, the ROC curves and AUC values of all models are generally consistent with the scores for H1, H2, and L. The incorporation of CF and LOWESS improves model performance, with all models except RandNet showing marked enhancement. Notably, CF-WBiGAN-GP achieves a higher score than CF-One-Class SVM but yields a lower AUC value. This discrepancy arises because the score reflects the numerical reasonableness of predictions, whereas AUC measures the ranking discrimination between samples. As shown in Table 4, for the high-susceptibility areas (label = 1), CF-WBiGAN-GP yields a mean score of 0.636, versus 0.102 for low-susceptibility areas (label = 0); the corresponding values for CF-One-Class SVM are 0.592 and 0.471. Clearly, CF-WBiGAN-GP performs better overall, yet it makes more misclassifications, meaning that some low-susceptibility samples receive higher scores than high-susceptibility ones. If more susceptibility classes (e.g., 3–5) are defined artificially, CF-WBiGAN-GP would achieve a higher AUC than CF-One-Class SVM. Thus, the two metrics are complementary.

#### 3.3.2. Visual Comparison in Three Representative Areas

**Figure 8:**
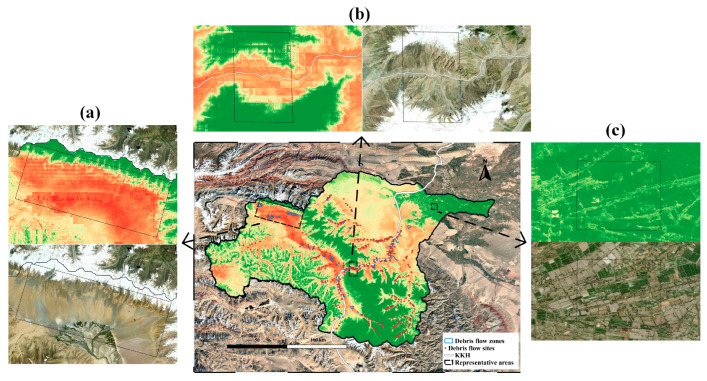
The visual comparison between the glacial debris flow susceptibility and high spatial resolution image from Google Earth in three representative areas. (**a**) a high-susceptibility zone along the glacial front; (**b**) a representative section of the KKH; (**c**) a low-susceptibility urban area.

Figure 8 demonstrates the strong predictive capability of the CF-WBiGAN-GP model, which accurately identifies both the debris flow zones from the training set and the debris flow sites from the external validation set. The model effectively captures the overall spatial distribution pattern of debris flows. It should be noted that the sites within this external validation set, located at the ends of debris flow channels, may not be appropriately assessed using the highest SI values [28].

The figure presents three representative areas extracted from the susceptibility map generated by the CF-WBiGAN-GP model: (a) a high-susceptibility zone along the glacial front, (b) a representative section of the KKH, and (c) a low-susceptibility urban area. In area (a), the model performs notably well, delineating hierarchical risk zonation with spatial differentiation. Area (b), located near the KKH, shows generally reasonable alignment with known hazardous areas. However, the model exhibits limitations in fully segmenting channel patterns, with noticeable omissions and inaccuracies along channel boundaries [29,30]. Area (c), covering an urbanized area identified as a low-susceptibility zone, is broadly correctly classified. Nonetheless, the result reflects a common issue in remote sensing semantic segmentation: localized decision confusion, indicating the model’s limited capacity for spatial understanding in context [31].

## 4. Discussion

### 4.1. Strength of Association Between DFPFs and Susceptibility

**Figure 9:**
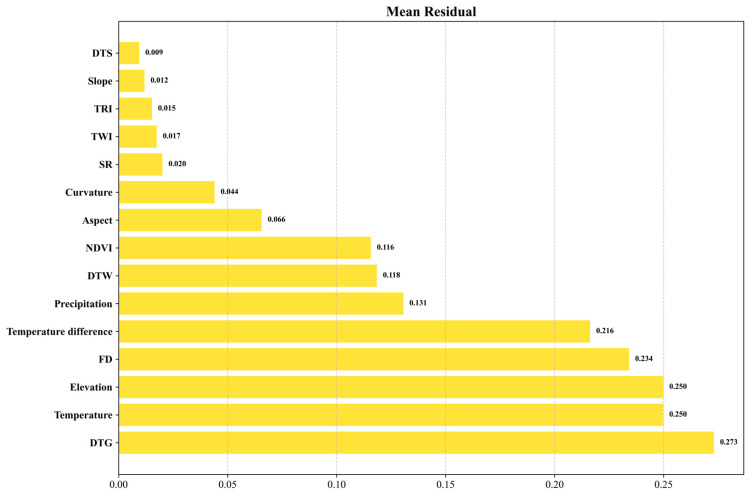
Mean residual of 15 DFPFs.

The strength of the association between DFPFs and GDF susceptibility is shown in Figure 9, quantified as the mean absolute residual: the sum of absolute differences between raw and smoothed CF values divided by the number of bins. This is not about the factor’s importance to the GDF initiation mechanism, but rather whether it can directly indicate susceptibility. When the raw CF values closely align with the LOWESS-smoothed curve, it indicates a strong relationship with susceptibility and minimal interference from other DFPFs. For example, slope ranks high in Figure 9; as seen in Figure 5, high CF values occur at slope angles around 10°, suggesting that high susceptibility can be inferred with high confidence at these slopes. In contrast, DTG ranks low in Figure 9, and thus its individual indication of susceptibility is less reliable.

DTS, slope, TRI, TWI, and SR show strong associations with GDF susceptibility. Consistent with previous studies [6,14,32], our findings further corroborate that topographic factors play a predominant role in debris flow susceptibility. The high rankings of slope and topographic indices (TRI, TWI) underscore the critical importance of terrain geometry in controlling material mobility and hydrological convergence [33]. Among all DFPFs, DTS ranks as the most distinguishable, as loose material is almost unlimited in this environment, and GDFs are easily triggered in gullies when seasonal rainfall and meltwater from rising temperatures occur. Meanwhile, SR ranks high, indicating that debris flows in the Gaizi basin are glacier-related. In contrast, DTG and Precipitation rank low. Although they are direct triggers of GDFs, they are unreliable for inferring susceptibility: glaciers vary in size, so a fixed distance to glacier does not necessarily correspond to meltwater input, and debris flow gullies can receive the same rainfall as nearby flat terrain. Differences in data scale may also contribute to this issue. Specifically, a 1 km monthly precipitation and temperature product was interpolated onto a 30 m grid; this limitation likely explains why precipitation ranks low. Consequently, the CF calculation for these factors may be unstable, as locations with the same DTG and precipitation can have different environments.

**Figure 10:**
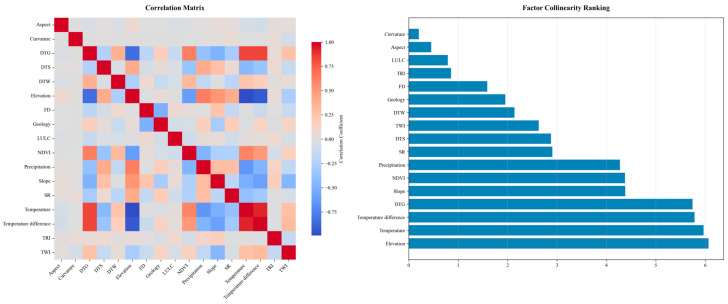
The correlation matrix and ranking between DFPFs.

To ensure the independence of each factor and to compare with the mean residual, we employed Pearson’s correlation coefficient to quantify the correlation between factors (Figure 10). The value of correlation coefficient (r) is within the range of [−1, 1] and the factor collinearity ranking score (R) is calculated by Equation (8). Overall, the trends of the factor collinearity ranking and the mean residual are consistent, especially at lower ranks. DFPFs with weak associations with other factors are statistically unaffected by them, making them reliable indicators of GDF susceptibility. However, factor collinearity is limited to comparisons within groups of 17 DFPFs and does not specifically address GDF occurrence, whereas the mean residual overcomes these limitations by utilizing historic inventory data and the CF.

(8) $$R_{j}=\sum_{i=1}^{17}|r(x_{i},x_{j})|-1$$

### 4.2. Advantages and Limitations of the Hybrid Anomaly Detection Models

This study demonstrated hybrid models for glacial debris flow susceptibility assessment. The DFPFs were first transformed into Certainty Factor (CF) values, which not only establish a statistical relationship with debris flow occurrence but also normalize the various factor measurements onto a uniform dimensionless scale. The Locally Weighted Scatterplot Smoothing (LOWESS) technique was then applied to convert the initially incomplete and outlier-prone CF distributions into continuous, idealized curves, effectively mitigating data noise and gaps [17]. Subsequently, both the single-class labeled data from the debris flow inventory and the processed CF values were incorporated into anomaly detection models to derive SI. Instead of relying on different classification methods, optimal susceptibility thresholds were determined by evaluating model performance across multiple evaluation metrics [34]. Validation using high/low susceptibility zones and an external inventory confirmed the robustness of the proposed models. Furthermore, the method’s relatively low data requirements render it particularly suitable for large-scale susceptibility analysis [35].

However, the single-class anomaly detection method also exhibits inherent limitations. The systematic tendency toward overestimating susceptibility reflects the fundamental challenge of one-class learning, where models trained exclusively on positive samples develop heightened sensitivity to potential hazards at the expense of specificity in stable area identification [36,37]. CF-WBiGAN-GP’s balanced performance may be attributed to its unique adversarial training. Unlike other models that typically excel at identifying high susceptibility but perform poorly in low-susceptibility recognition [38], GAN’s generator provides quasi-multi-class training capabilities from single-class inputs by generating pseudo-samples. This architecture enables more balanced performance and mitigates the challenge of limited training data through its generative adversarial mechanism.

## 5. Conclusions

This study developed a hybrid modeling framework integrating statistical methods (CF, LOWESS) and machine learning methods (L2 Norm, One-Class SVM, Isolation Forest, RandNet, WBiGAN-GP) for debris flow susceptibility assessment in the Gaizi River Basin, a high-altitude glacial environment traversed by the strategic Karakoram Highway. The CF was transformed to continuous distributions using LOWESS. The DFPFs’ association with susceptibility was determined using mean residuals between raw and smoothed CF curves. The susceptibility map generated by the hybrid model overcomes the limitations of traditional classification-based methods.

Model validation incorporated both high-susceptibility areas from the external debris flow inventory and low-susceptibility areas from stable landscapes, providing rigorous performance evaluation across the susceptibility spectrum. Further analysis through three representative areas demonstrated the model’s capability in identifying debris flow components while revealing limitations in complex boundary delineation.

The analysis of the strength of association between DFPFs and susceptibility revealed a clear hierarchy of predisposing factors, with distance to stream (DTS), slope, and topographic indices (TRI, TWI) emerging as the factors most strongly associated with susceptibility. This quantitative ranking aligns with established principles, highlighting the dominance of topographic factors in controlling debris flow initiation. The relatively lower ranking of precipitation and DTG reflects the fact that glaciers vary in size and shape, making meltwater input difficult to infer, and that areas without debris flows can experience rainfall comparable to that of high-susceptibility zones, thus these factors alone cannot reliably indicate susceptibility.

This research provides a methodology with low label requirements for susceptibility assessment in high-mountain environments, with particular relevance for the China–Pakistan Economic Corridor. Future work should focus on integrating process-based physical models to better understand glacier-related debris flow initiation mechanisms, while incorporating temporal dynamics and triggering thresholds for improved early warning capabilities.

## Figures and Tables

**Table 1:** Datasets used in this study.

Datasets	Source	Type	Scale
DEM	ASTER DEM	Grid	30 m
Geological data	National 1:1,000,000 Geological Map Spatial Database [10]	Vector	1: 1,000,000
Land use	The 30 m annual land cover dataset and its dynamics in China from 1990 to 2019 [11]	Grid	30 m
Meteorological data	1 km monthly temperature and precipitation dataset for China from 1901 to 2017 [12]	NETCDF	1000 m
Glacier distribution	The second glacial inventory data set of China (v1.0) [13]	Vector	-
Debris flow inventory	Remote sensing interpretation and a dataset of distributions and characteristics of debris flows in the China–Pakistan Economic Corridor [1]	Vector	-

**Table 2:** Experimental parameters of LOWESS.

DFPFs	Bins	Bandwidth	Iteration
Aspect	200	60	1
Curvature	100	10	1
DTG	200	60	3
DTS	100	10	1
DTW	100	50	1
Elevation	100	50	1
FD	100	70	2
Geology	36	-	-
LULC	8	-	-
NDVI	75	15	1
Precipitation	50	20	2
Slope	100	10	1
Solar radiation	100	10	1
Temperature	100	30	6
Temperature difference	100	30	6
TRI	100	10	1
TWI	100	10	1

**Table 3:** The main parameters used in 5 models.

Methods	Strategy	Main Hyperparameters
L2 Norm	distance	-
One-Class SVM	density	Kernel = ‘rbf’, gamma = ‘scale’, nu = 0.1
iForest	ensemble	Contamination = 0.1, random_state = 42
RandNet	ensemble	encoding_dim = 32, n_estimators = 21, learning rate = 0.001
WBiGAN-GP	GAN	latent_dim = 51, lambda_gp = 10, learning rate = 0.0001

**Table 4:** H1, H2, and L metrics for all models.

Models	H1	H2	L
L2 Norm	7.88	7.07	2.90
CF-L2 Norm	8.01	6.30	7.06
One-Class SVM	7.28	8.31	2.23
CF-One-Class SVM	5.92	4.71	8.73
iForest	7.75	5.70	6.64
CF-iForest	8.68	7.47	4.37
RandNet	8.25	7.47	4.77
CF-RandNet	8.49	7.42	4.05
WBiGAN-GP	3.23	3.45	6.62
CF-WBiGAN-GP	8.39	6.36	8.98

## Data Availability

The source code is available at the link: https://github.com/yique258/Susceptibility-assessment-using-different-hybrid-anomaly-detection-models (accessed on 15 June 2026).

## References

[1] Jiang Y., Guo Y., He J. (2021). A Dataset of Distributions and Characteristics of Debris Flows in the China-Pakistan Economic Corridor. China Sci. Data.

[2] Jiang R., Zhang L., Lu W., Peng D., He X., Xiao S., Wei M. (2026). A Numerical Model for Cascading Glacier Mass Flow Analysis (Gmfa): Erosion-Deposition Dynamics, Phase Changes, and Multi-Hazard Chain Transformations. J. Rock. Mech. Geotech. Eng..

[3] Jiang N., Su F., Li Y., Guo X., Zhang J., Liu X. (2021). Debris Flow Assessment in the Gaizi-Bulunkou Section of Karakoram Highway. Front. Earth Sci..

[4] Huang F., Cao Z., Guo J., Jiang S.-H., Li S., Guo Z. (2020). Comparisons of Heuristic, General Statistical and Machine Learning Models for Landslide Susceptibility Prediction and Mapping. CATENA.

[5] Guo Z., Zeng T., Zhang Y., Yu W., Wang L., Guo Z., Glade T. (2025). A Novel Hybrid Model Integrating High Resolution Remote Sensing and Stacking Ensemble Techniques for Landslide Susceptibility Mapping: Application to Event-Based Landslide Inventory. Geomorphology.

[6] Yu R., Guo R., Jiang L., Shao Y., Zhou Z. (2024). Susceptibility Assessment of Glacier-Related Debris Flow on the Southeastern Tibetan Plateau Using Different Hybrid Machine Learning Models. Sci. Total Environ..

[7] Li K., Zhao J., Chen G., Li Y. (2025). Debris-Flow Susceptibility Assessment Using Deep Learning Algorithms with Geodetector for Factor Optimization. Bull. Eng. Geol. Environ..

[8] Qing F., Zhao Y., Meng X., Su X., Qi T., Yue D. (2020). Application of Machine Learning to Debris Flow Susceptibility Mapping Along the China–Pakistan Karakoram Highway. Remote Sens..

[9] Lin K., Jiapaer G., Yu T., Zhang L., Liang H., Chen B., Ju T. (2024). Identification of Potential Landslides in the Gaizi Valley Section of the Karakorum Highway Coupled with Ts-Insar and Landslide Susceptibility Analysis. Remote Sens..

[10] Pang J., Ding X., Han K., Zeng Y., Chen A., Zhang Y., Zhang Q., Yao D. (2017). The National 1: 1,000,000 Geological Map Spatial Database. Geol. China.

[11] Yang J., Huang X. (2021). The 30 M Annual Land Cover Dataset and Its Dynamics in China from 1990 to 2019. Earth Syst. Sci. Data.

[12] Peng S., Ding Y., Liu W., Li Z. (2019). 1 Km Monthly Temperature and Precipitation Dataset for China from 1901 to 2017. Earth Syst. Sci. Data.

[13] Liu S., Guo W., Xu J. (2021). The Second Glacial Catalogue Data Set of China (V1.0).

[14] Liu Y., Chen J., Sun X., Li Y., Zhang Y., Xu W., Yan J., Ji Y., Wang Q. (2024). A Progressive Framework Combining Unsupervised and Optimized Supervised Learning for Debris Flow Susceptibility Assessment. CATENA.

[15] Liu R., Chang C., Zhong R., Lu S. (2025). Soil Moisture Monitoring Method and Data Products: Current Research Status and Future Development Trends. Remote Sens..

[16] Wang Q., Guo Y., Li W., He J., Wu Z. (2019). Predictive Modeling of Landslide Hazards in Wen County, Northwestern China Based on Information Value, Weights-of-Evidence, and Certainty Factor. Geomat. Nat. Hazards Risk.

[17] Zhao H., Chen W., Zhang C., Kang D. (2023). Rapid Estimation of Seismic Intensities by Analyzing Early Aftershock Sequences Using the Robust Locally Weighted Regression Program (Lowess). Nat. Hazards Earth Syst. Sci..

[18] Ruff L., Vandermeulen R., Goernitz N., Deecke L., Siddiqui S.A., Binder A., Müller E., Kloft M. Deep One-Class Classification. Proceedings of the 35th International Conference on Machine Learning.

[19] Que Z., Lin C.J. (2025). One-Class Svm Probabilistic Outputs. IEEE Trans. Neural Netw. Learn. Syst..

[20] Liu F.T., Ting K.M., Zhou Z.H. (2008). Isolation Forest. ICDM ’08: Proceedings of the 2008 Eighth IEEE International Conference on Data Mining.

[21] Yepmo V., Smits G., Lesot M.J., Pivert O. (2024). Leveraging an Isolation Forest to Anomaly Detection and Data Clustering. Data Knowl. Eng..

[22] Chen J., Sathe S., Aggarwal C., Turaga D. (2017). Outlier Detection with Autoencoder Ensembles. Proceedings of the 2017 Siam International Conference on Data Mining.

[23] Donahue J., Krähenbühl P., Darrell T. (2016). Adversarial Feature Learning. https://ui.adsabs.harvard.edu/abs/2016arXiv160509782D.

[24] Gulrajani I., Ahmed F., Arjovsky M., Dumoulin V., Courville A.C. (2017). Improved Training of Wasserstein Gans. https://ui.adsabs.harvard.edu/abs/2017arXiv170400028G.

[25] Heusel M., Ramsauer H., Unterthiner T., Nessler B., Hochreiter S. Gans Trained by a Two Time-Scale Update Rule Converge to a Local Nash Equilibrium. Proceedings of the Advances in Neural Information Processing Systems 30 (NIPS 2017).

[26] Dai Z., Zhao L., Wang K., Zhou Y. (2024). Mode Standardization: A Practical Countermeasure against Mode Collapse of Gan-Based Signal Synthesis. Appl. Soft Comput..

[27] Ren Y., Zhang Y., Kang J. (2019). A Dataset of Glacier and Glacial Lake Distribution in Key Areas of the China-Pakistan Economic Corridor during 2013–2017. China Sci. Data.

[28] Ning L., Hu K., Li P., Cheng H., Liu S., Zhang Q. (2025). Peak Discharge Amplication of Debris Flows in Colluvial Channels with Varying Cross-Sections. Environ. Earth Sci..

[29] Maslov K.A., Persello C., Schellenberger T., Stein A. (2025). Globally Scalable Glacier Mapping by Deep Learning Matches Expert Delineation Accuracy. Nat. Commun..

[30] Zhou C., Gan L., Cao Y., Wang Y., Segoni S., Shi X., Motagh M., Singh R.P. (2025). Landslide Susceptibility Assessment of the Wanzhou District: Merging Landslide Susceptibility Modelling (Lsm) with Insar-Derived Ground Deformation Map. Int. J. Appl. Earth Obs. Geoinf..

[31] Zhang T., Li X., Fei H., Yuan H., Wu S., Ji S., Change Loy C., Yan S. (2024). Omg-Llava: Bridging Image-Level, Object-Level, Pixel-Level Reasoning and Understanding. https://ui.adsabs.harvard.edu/abs/2024arXiv240619389Z.

[32] Huang F., Ye Z., Jiang S.-H., Huang J., Chang Z., Chen J. (2021). Uncertainty Study of Landslide Susceptibility Prediction Considering the Different Attribute Interval Numbers of Environmental Factors and Different Data-Based Models. CATENA.

[33] Fang Z., Wang Y., van Westen C., Lombardo L. (2024). Landslide Hazard Spatiotemporal Prediction Based on Data-Driven Models: Estimating Where, When and How Large Landslide May Be. Int. J. Appl. Earth Obs. Geoinf..

[34] Youssef A.M., Pourghasemi H.R. (2021). Landslide Susceptibility Mapping Using Machine Learning Algorithms and Comparison of Their Performance at Abha Basin, Asir Region, Saudi Arabia. Geosci. Front..

[35] Chen S., Miao Z., Wu L., He Y. (2020). Application of an Incomplete Landslide Inventory and One Class Classifier to Earthquake-Induced Landslide Susceptibility Mapping. IEEE J. Sel. Top. Appl. Earth Obs. Remote Sens..

[36] Leevy J.L., Hancock J., Khoshgoftaar T.M., Zadeh A.A. (2023). One-Class Classifier Performance: Comparing Majority Versus Minority Class Training. 2023 IEEE 35th International Conference on Tools with Artificial Intelligence (ICTAI).

[37] Zhu A.X., Miao Y., Yang L., Bai S., Liu J., Hong H. (2018). Comparison of the Presence-Only Method and Presence-Absence Method in Landslide Susceptibility Mapping. CATENA.

[38] Jia Z., Cheng Z., Chang Z., Li Q., Huang F., Peng Y. (2026). Non-Landslide Sample for Landslide Susceptibility Prediction Modeling: A Review of Selection Strategies and Their Influence Rules. J. Rock. Mech. Geotech. Eng..

